# Spatiotemporal gait assessment as an indicator of patient satisfaction after arthroscopic partial meniscectomy

**DOI:** 10.1007/s00508-025-02698-1

**Published:** 2026-01-19

**Authors:** Ildikó Morochovičová, Radoslav Hreha, Rastislav Burda, Radoslav Morochovič

**Affiliations:** 1 Department of Physiatry, Balneology, and Medical Rehabilitation, Faculty of Medicine, P.J.Šafárik University and University Hospital of L. Pasteur, Rastislavova 43, 04190 Košice, Slovakia; 2 Department of Trauma Surgery, Faculty of Medicine, P.J.Šafárik University and University Hospital of L. Pasteur, Rastislavova 43, 04190 Košice, Slovakia

**Keywords:** Knee surgery, Wearable sensors, Gait analysis, Postoperative recovery, Outcome

## Abstract

**Background:**

Arthroscopic partial meniscectomy is one of the most commonly performed orthopedic procedures, yet many patients remain dissatisfied in the early postoperative period. Whether dissatisfaction is reflected in changes in spatiotemporal gait parameters remains unclear but such information could help the early identification of patients at risk of poor outcomes. This prospective observational cohort study aimed to assess gait parameters in patients after partial meniscectomy using foot-worn wearable sensors and to evaluate their potential as indicators of postoperative satisfaction.

**Methods:**

In this study 27 patients scheduled for partial meniscectomy were prospectively enrolled; the final group comprised 22 patients who completed all assessments. Gait parameters were measured preoperatively and at 1 and 2 months postoperatively. At final follow-up, patients rated their satisfaction. Patients were categorized as satisfied (*n* = 18) or dissatisfied (*n* = 4).

**Results:**

Significant improvements in stride length (*p* < 0.01) and walking speed (*p* = 0.01) were observed at 2 months postoperatively in the entire group. Among satisfied patients, both stride length and walking speed significantly improved (*p* < 0.01), whereas in the dissatisfied subgroup cadence and walking speed did not return to baseline values. Patient satisfaction was best predicted by an increase in cadence, with a positive predictive value of 93% (95% confidence interval, CI 72–99%).

**Conclusion:**

Patients who reported satisfaction demonstrated improvements in gait parameters, with cadence emerging as the strongest predictor of postoperative satisfaction.

## Introduction

The prevalence of meniscus tears increases with age and from middle age onwards they are predominantly degenerative and often associated with knee osteoarthritis [1, 2]. Treatment of acute meniscus tears in younger patients with greater size and tear location in the vascularized part of the meniscus is arthroscopic meniscus repair [3]. Otherwise, surgical treatment consists of arthroscopic partial meniscectomy (APM) or initial management with physical therapy alone, with crossover to APM if therapy fails [3–5]. As a result, over the last decades APM has become one of the most frequently performed orthopedic procedures [5, 6]. Following APM, patients can present with abnormalities in limb motion and muscle activation patterns during locomotor activities as well as pain, effusion and a loss of quadriceps muscle strength [7, 8]. Therefore, physical therapy is usually prescribed, either as supervised outpatient sessions, unsupervised home exercise programs, or a combination of both, with the goal of facilitating a return to normal daily activities and, in some cases, sports within 2–8 weeks [3, 8, 9]. The course of postoperative recovery is monitored through regular follow-ups, including assessment of objective surgically relevant outcomes (e.g., range of motion), patient-reported outcome measures (PROMs), and patient-reported satisfaction, with the latter often representing the most important measure from the patient’s perspective [10–12]. For routine follow-up assessments, certain complementary objective measurements, such as stabilometry and spatiotemporal (ST) gait analysis, were previously available only in specialized laboratories, which limited their practical use [13–15]. With the recent availability and advancement of wearable sensors, demonstrated to be as precise and accurate as laboratory-based methods, their application can now be extended to standard medical examinations and may enable objective remote monitoring of postoperative gait function [14, 16]. Aside from their role in monitoring, it remains unclear whether ST gait parameters can help distinguish between unfavorable and favorable courses in the early postoperative period. This study aimed to assess longitudinal within-patient changes in ST gait parameters after APM using foot-worn wearable sensors and, in an exploratory secondary analysis, to identify which postoperative ST changes best discriminate a favorable postoperative course.

## Methods

This prospective observational cohort study included all symptomatic adult patients (≥ 18 years) scheduled for arthroscopic surgery for magnetic resonance imaging (MRI)-confirmed meniscal injury of the knee joint between April 2023 and April 2024. Only patients who underwent APM and who provided informed consent were included in longitudinal analysis. Exclusion criteria included any previously diagnosed neurological, orthopedic, rheumatological or oncological condition, balance disorder or history of lower limb injury treated within the past 6 months.

**Fig. 1:**
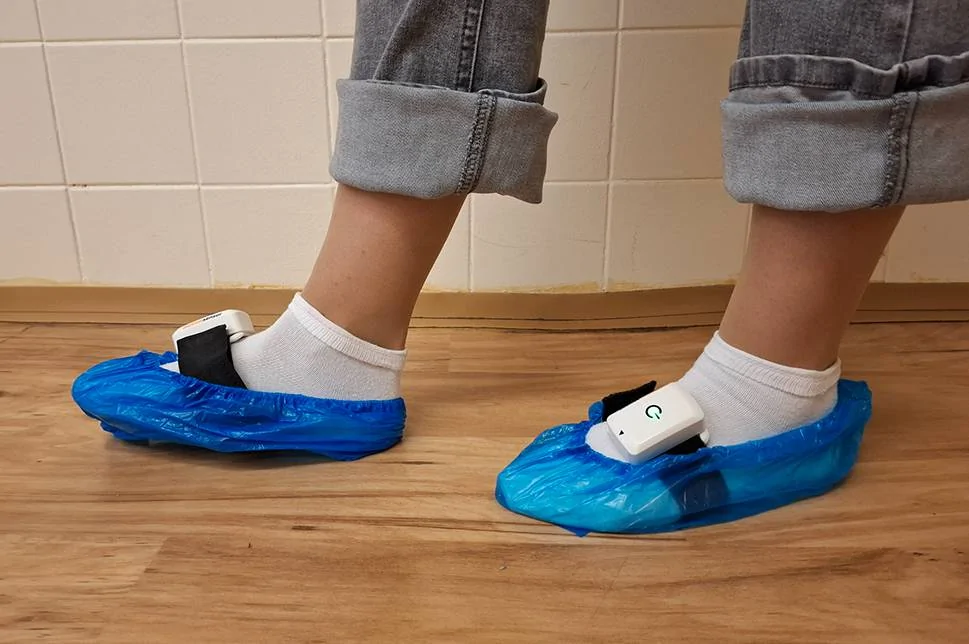
Sensors mounted on the dorsum of each foot

Baseline assessment (≤ 1 day preoperatively) included anthropometric measurements, demographic data and patient-reported knee status on a 0–100 numeric rating scale (NRS) where 0 indicated a completely non-functional knee joint and 100 represented a fully functional/ideal knee condition. Gait parameters were recorded using two PABLO® MotionSensors (Tyromotion GmbH, Graz, Austria), which measured angular velocity and acceleration at a sampling frequency of 110 Hz. Sensors were attached to the back of each foot (Fig. 1) and participants completed a single 10m straight walk at a self-selected comfortable walking speed [14]. Sensor data were transmitted wirelessly to a computer, processed using tyroS software, version 6.1 (Tyromotion GmbH) and the resulting values were exported as tabulated numerical data in pdf format. The mean values of the spatiotemporal gait parameters of interest (stride length, cadence and walking speed) were used for analysis.

Patients were reassessed at 1 month and at 2 months postoperatively. At the final follow-up, patients reported their satisfaction with the postoperative knee condition on a 15-point scale ranging from −7 to +7. A score of −7 indicated strong dissatisfaction, 0 indicated neutrality and +7 indicated complete satisfaction. Patients with scores > 0 were classified as satisfied (Sat), while those with scores ≤ 0 were classified as dissatisfied (Dissat). The local ethics committee reviewed and approved the study protocol (2023/EK/05022).

### Statistical methods

Data for age, weight, height, body mass index (BMI) and knee status are presented as mean with standard deviation (SD). Normality was assessed using the Shapiro-Wilk test. Between-group differences (Sat vs. Dissat) were evaluated using Student’s *t*-test for independent samples or the Mann-Whitney U test, as appropriate. Changes in gait parameters over time within the entire cohort (All group) and within subgroups (Sat and Dissat) were assessed using paired t‑test or Wilcoxon signed-rank test, according to data distribution. Confidence interval calculations were performed using standard statistical procedures implemented in cited MedCalc® software, specifically the Clopper–Pearson exact method for sensitivity and specificity and logit-based confidence intervals for predictive values as described by Mercaldo. Statistical significance was set at *p* < 0.05. All analyses were performed using MedCalc® software (version 23.1.3; Ostend, Belgium).

## Results

A total of 27 patients were enrolled and 5 did not attend at least 1 follow-up visit. Of the 22 patients who completed the study (All group), 18 reported satisfaction with the treatment outcome at final follow-up (Sat) subgroup, and 4 were dissatisfied (Dissat) subgroup. No statistically significant differences in baseline characteristics were found between the Sat and Dissat subgroups, although a trend toward a higher BMI was observed in the Sat subgroup (*p* = 0.05, Table 1).

**Table 1:** Baseline characteristics of the entire cohort (All_0_), satisfied subgroup (Sat_0_) and dissatisfied subgroup (Dissat_0_). The *p*-values indicate comparisons between Sat₀ and Dissat₀. Data are presented as mean (standard deviation) unless otherwise specified

		All_0_ (*n* = 22)	Sat_0_ (*n* = 18)	Dissat_0_ (*n* = 4)	*p*
Age (years)		46.9 (11.5)	46.7 (6.0)	47.5 (12.6)	0.91
Sex	Men (*n*)	11	9	2	1.00
	Women (*n*)	11	9	2	
Weight (kg)		84.5 (16.3)	86.2 (17.0)	76.8 (11.1)	0.31
Height (m)		1.72 (0.10)	1.71 (0.10)	1.76 (0.13)	0.38
Body mass index (kg/m^2^)		28.7 (6.1)	29.6 (6.3)	24.8 (3.1)	0.05
Self-rated knee condition (0–100; worst-best)		57.6 (20.5)	58.2 (18.9)	55.0 (30.0)	0.78
Stride length (cm)		115 (17.4)	115 (16.4)	115 (24.0)	0.98
Cadence (steps/min)		107 (15.4)	106 (16.2)	115 (8.9)	0.29
Speed (km/h)		3.7 (0.6)	3.6 (0.6)	3.9 (0.6)	0.40

During postoperative follow-up, all monitored ST gait parameters in the All group showed a gradual increase, with statistically significant improvements in stride length and walking speed observed at 2 months postoperatively (Table 2).

**Table 2:** Change of mean spatiotemporal gait parameters in the entire cohort (All, *n* = 22)

	All_0_	All_1_	All_2_	*p*
Stride length (cm)	115 (17)	119 (17)	126 (15)	0.32
				< 0.01*
Cadence (steps/min)	107 (15)	109 (24)	118 (27)	0.93
				0.14
Speed (km/h)	3.7 (0.6)	3.9 (1.3)	4.5 (1.2)	0.42
				0.01*

*All*_*0*_ baseline (preoperative), *All*_*1*_ 1 month, *All*_*2*_ 2 months postoperatively. In the *p* column, the upper value indicates All_0_ vs. All_1_, and the lower value All_0_ vs. All_2_. Values are means with standard deviation (SD). *p* probability; * = statistically significant difference.

In the Sat subgroup, statistically significant increases were observed in stride length and walking speed, with a nonsignificant trend toward increased cadence at final follow-up (Table 3). In contrast, the Dissat subgroup demonstrated no improvement in stride length and showed reductions in cadence and walking speed.

**Table 3:** Change of mean spatiotemporal gait parameters in satisfied (Sat, *n* = 18) and dissatisfied (Dissat, *n* = 4) subgroups

	Sat_0_	Sat_1_	Sat_2_	*p*	Dissat_0_	Dissat_1_	Dissat_2_	*p*
Stride length (cm)	115 (16)	122 (16)	128 (13)	0.04	115 (24)	105 (19)	116 (20)	0.42
				< 0.01*				0.94
Cadence (steps/min)	105 (16)	113 (24)	121 (28)	0.27	115 (9)	91 (13)	102 (19)	0.07
				0.06				0.21
Speed (km/h)	3.6 (0.6)	4.1 (1.2)	4.7 (1.2)	0.07	3.9 (0.6)	2.9 (0.9)	3.6 (1.1)	0.14
				< 0.01*				0.67

*Sat*_0_/Dissat_0_ baseline (preoperative), *Sat*_*1*_*/Dissat*_*1*_ 1 month, *Sat*_*2*_*/Dissat*_*2*_ 2 months postoperatively. In the *p* column, the upper value indicates Sat_0_ vs. Sat_1_, (or Dissat_0_ vs. Dissat_1_) and the lower value Sat_0_ vs. Sat_2_ (or Dissat_0_ vs. Dissat_2_). Values are means with standard deviation (SD). *p* probability; * = statistically significant difference

An increase in cadence between baseline and final measurement was observed in 14 of 18 patients (78%) in the Sat subgroup, whereas cadence did not increase in 3 of 4 patients (75%) in the Dissat subgroup. Other ST gait parameters followed a similar pattern, increasing in most Sat patients and not increasing in 25–50% of Dissat patients.

Improvement in ST gait parameters predicted treatment satisfaction in 82–93% of cases, with cadence showing the highest predictive value and acceptable discrimination ability (area under the curve, AUC 0.76; 95% CI: 0.54–0.92) (Table 4).

**Table 4:** Sensitivity and specificity of spatiotemporal gait parameters in distinguishing between satisfied (Sat) and dissatisfied (Dissat) groups. Values are given as counts

		Sat (*n* = 18)	Dissat (*n* = 4)	Sensitivity (95% CI) %	Specificity (95% CI)	Positive predictive value (95% CI)	AUC ROC (95% CI)
Stride length	Incr	14	3	78 (52–94)	25 (1–81)	82 (72–90)	0.51 (0.29–0.73)
	Noincr	4	1				
Cadence	Incr	14	1	78 (52–94)	75 (19–99)	93 (72–99)	0.76 (0.54–0.92)
	Noincr	4	3				
Speed	Incr	14	2	78 (52–94)	50 (7–93)	88 (72–95)	0.64 (0.41–0.83)
	Noincr	4	2				

*CI* confidence intervals. Increase (*Incr*)/no increase (*Noincr*) values 2 months after surgery compared to baseline values. *AUC ROC* area under the receiver operating characteristic curve.

## Discussion

In our study, during the early postoperative period, we observed significant improvements in mean stride length and walking speed in the All group following APM. These findings are in line with results from previous studies [15, 17]. Karahan et al. (2022) noted increases in step length, cadence and velocity which were no longer significantly different from those of healthy controls at 12 weeks after APM [15]. Similarly, Li et al. (2020) reported significant gains in walking speed and stride length, with a nonsignificant trend toward increased cadence at 3 months postoperatively [17].

Although these increases of mean in ST parameters point to patients’ early postoperative recovery, up to half of them perceived the knee symptoms as unacceptable after APM. Pedersen et al. documented that among 614 patients undergoing arthroscopic knee surgery (95% after APM), only 45% reported satisfactory symptom levels at 3‑month follow-up [18].

In the present study, treatment satisfaction with the postoperative knee condition was reported in 18 of 22 patients (82%). At baseline, the Sat subgroup did not differ from the Dissat subgroup in self-rated knee condition, anthropometric parameters, or ST gait parameters. In the Sat subgroup, ST parameters exhibited an improvement as early as the first follow-up, with significant increases from baseline to final assessment in both mean stride length and walking speed, with cadence showing a nonsignificant upward trend. In contrast, the Dissat subgroup showed worsening of mean ST gait parameters at the first follow-up. At the final assessment, cadence and walking speed still failed to reach preoperative values, while stride length only returned to its baseline value. An increase in cadence between baseline and the final measurement was observed in 15 patients, 14 of whom were in the Sat subgroup. Thus, an increase in cadence emerged as the strongest indicator of patient satisfaction, with a positive predictive value of 93% (95% CI: 72%–99%). As reliable measurements of basic ST gait parameters can now be obtained using triaxial accelerometers in smartphones [19], incorporating postoperative monitoring of one or more ST parameters could enable early detection of trends in functional recovery. When negative trends are identified, i.e., no increase in ST values above baseline as early as the first and at the latest by the second postoperative month, a proactive, individualized approach with timely adjustment of the postoperative rehabilitation plan may help improve functional outcomes and enhance patient satisfaction after APM.

A limitation of this study is the small sample size which prevented further stratification. Postoperative changes in ST gait parameters may have been influenced by additional uncontrolled factors, including individual compliance with the rehabilitation program, the use of analgesics or intra-articular glucocorticoid injections during the recovery period. The Dissat subgroup with only four patients led to wide confidence intervals around specificity and AUC estimates of the assessed ST gait parameters. As a result, the discriminative values reported here should be regarded as hypothesis-generating and interpreted with caution. Future research with larger cohorts is needed to validate these findings to clarify the predictive value of ST gait parameters. Such studies could enhance the body of evidence and may support the consideration of simple gait monitoring systems for potential integration into routine clinical practice.

## Conclusion

Our findings suggest that during the early postoperative period following APM, patients who report satisfaction with the postoperative knee condition exhibit improvements in ST gait parameters. Among these parameters, an increase in cadence demonstrated the highest predictive value for patient satisfaction. Monitoring ST gait parameters, particularly cadence, using wearable sensors may offer a simple and objective approach for the early identification of patients at risk of poor recovery, thereby enabling timely intervention and potentially improving outcomes.
